# Environmental drivers of bat activity at high latitudes

**DOI:** 10.1186/s12862-026-02497-w

**Published:** 2026-02-07

**Authors:** Thomas M. Lilley, Eero J. Vesterinen, Ville V. Vasko, Anna S. Blomberg, Mari Aas Fjelldal

**Affiliations:** 1 Finnish Museum of Natural History, University of Helsinki, Helsinki, Finland; 2 Department of Biology, University of Turku, Turku, Finland; 3 Turku University of Applied Sciences, Turku, Finland; 4 Norwegian University of Life Sciences, Environmental Sciences and Natural Resource Management, As, Norway

**Keywords:** Photoperiod, High-latitude ecosystems, Crepuscular, Nocturnality, Insectivore, Chiroptera

## Abstract

In high-latitude ecosystems, insectivorous bats must make use of short nights during the summer, during which temperature and light conditions interact in non-linear ways. These abiotic dynamics directly influence insect availability and, in turn, bat activity. We examined the influence of night-time temperature and night length on the nightly and seasonal activity patterns of the northern bat (*Eptesicus nilssonii*) across Finland (60°–66°N), using passive acoustic monitoring data collected over 9,000 detector-nights across seven years. We predicted that bat activity would be constrained by low temperatures, strong winds or heavy rain, and that the nightly pattern would depend on an interaction between night length and weather conditions. Our results show activity peaked 1–2 h after sunset under most conditions, with ambient temperature strongly influencing overall activity levels. On short, warm nights, activity was relatively evenly distributed across the few hours of darkness, whereas long, warm nights produced a bimodal activity pattern with peaks after sunset and before sunrise. This dual-peaked pattern occurred only under thermal conditions that likely support sustained insect activity. In contrast, on colder or windier nights, activity was sharply concentrated to the first hours after sunset. Seasonal and latitudinal trends revealed that activity was most restricted in spring, particularly in northern regions, while the progressing summer expressed more evenly distributed patterns. In autumn, activity patterns diverged across latitudes, reflecting interactions between temperature and night length. The results of our study demonstrate that *E. nilssonii* may dynamically adjust its foraging behaviour in response to interacting abiotic constraints, optimizing energy gain while minimizing predation risk. Our findings quantify the environmental thresholds facilitating various activity patterns and provide rare evidence of bimodal nocturnal activity at high latitudes. Our study highlights the importance of large-scale, long-term monitoring for understanding how changing climatic conditions influence species behaviour in boreal ecosystems.

**Supplementary Information:**

The online version contains supplementary material available at 10.1186/s12862-026-02497-w.

## Introduction

The summer is relatively short at northerly, sub-arctic latitudes compared to more southerly latitudes and is characterised by considerable variability in the amount of circadian light and thermal conditions across the season. The night is at its shortest length during summer solstice but mean diurnal temperatures do not reach their peak until later in the summer, at which point the night length has begun to increase again. Therefore, organisms need to be adapted to an environment in which temperature and photoperiod change rapidly but non-linearly. These same abiotic conditions influence ecosystem productivity in the boreal zone, and the utilization of the resources in these highly dynamic conditions can be challenging for nocturnal insectivorous consumers, such as bats (Chiroptera). This is because the activity of their insect prey rapidly declines with decreasing ambient temperature after sunset, and the overall limited foraging time during white summer nights, where the night never becomes truly dark, promotes a situation where resources may be available only for a limited time across an already constricted foraging period [1, 2].

Although nightly activity patterns have been described for many bat species across different habitats and reproductive states [2–10], the interaction between photoperiod and nighttime temperature on the nightly activity patterns of bats has not been studied in detail [although see 1, 12]. So far, it has been acknowledged that foraging activity of bats at high latitudes coincides with minimum light intensity [1, 11, 12]. This behaviour has been suggested as a response to avoid predation by avian predators [13–15], although thermal and energetic constraints of bats may also have played a role in promoting nocturnal activity [16, 17]. Given that aerial insect activity is most pronounced during the twilight periods of dusk and dawn [2, 11], bats in the boreal zone encounter their insect prey as their activity is already declining after sunset due to the constraints of their nocturnality [1].

At northerly latitudes, the abiotic conditions, particularly temperature and ambient light vary considerably across the active season of insectivorous bats. Their activity is governed by the dual constraints of energy input (insect availability) and energy output (thermoregulation). The activity of their insect prey declines after sunset: a phenomenon dictated by the inherent diel activity of insects [18, 19], and their response to decreasing temperature [1, 20–22]. Therefore, bats attempting to forage may be deprived of energy intake on colder nights. Insectivorous bats can overcome these energetic challenges through the use of energy-saving torpor on nights when foraging prospects are likely to be poor [20, 23–25]. Environmental conditions [26, 27], as well as species-specific ecology [12, 28] and the individual state [25, 29, 30] can dictate the overall activity of bats on a given night, as well the period during which they are active within a night. Therefore, activity and foraging behaviour in bats can be highly dynamic depending on prevailing ambient conditions [25].

Here we investigate the influence of night-time temperature and night length on the activity patterns of the Northern bat, *Eptesicus nilssonii* (Keyserling and Blasius, 1839), both within a night and across nights using a data set collected across Finland, spanning the active months of temperate bats (April-October) over seven years. We hypothesise that due to the thermal constraints [31] and diel patterns of insects [18, 32, 33], as well as the use of torpor by bats [25], bat activity on cold nights is low and limited to the period immediately following sunset. In contrast we hypothesise that on short mild or warm nights, bat activity increases with temperature to take advantage of insects that are available [34]. Furthermore, we use our empirical dataset to translate our model to actual scenarios over the active season of bats at varying latitudes across Finland.

## Materials and methods

### Study species

Here, we use data collected on the activity patterns of *E. nilssonii* to explore our hypothesis. The species holds the record for the most northerly breeding colony in Troms, Norway, at 69° N [35]. The distribution range of *E. nilssonii* spans the boreal zone from western Scandinavia to northern Japan, with some isolated, high-altitude populations in southerly locations representing glacial refugia [36]. The species is adapted to the short season of activity at northerly latitudes with parturition around summer solstice [37, 38], rapid development of pups [39], and fast accumulation of fat reserves in the autumn [30]. The species was selected because it is the only species with high enough abundance at the high latitudes of our northern-most study sites (see section below) to provide enough data for the statistical modelling our research questions require. Furthermore, the echolocation calls of *E. nilssonii* can be identified automatically identified in a robust manner, allowing us to process and analyse large amounts of acoustic data with confidence (e.g [28, 40]).

### Data collection

**Fig. 1:**
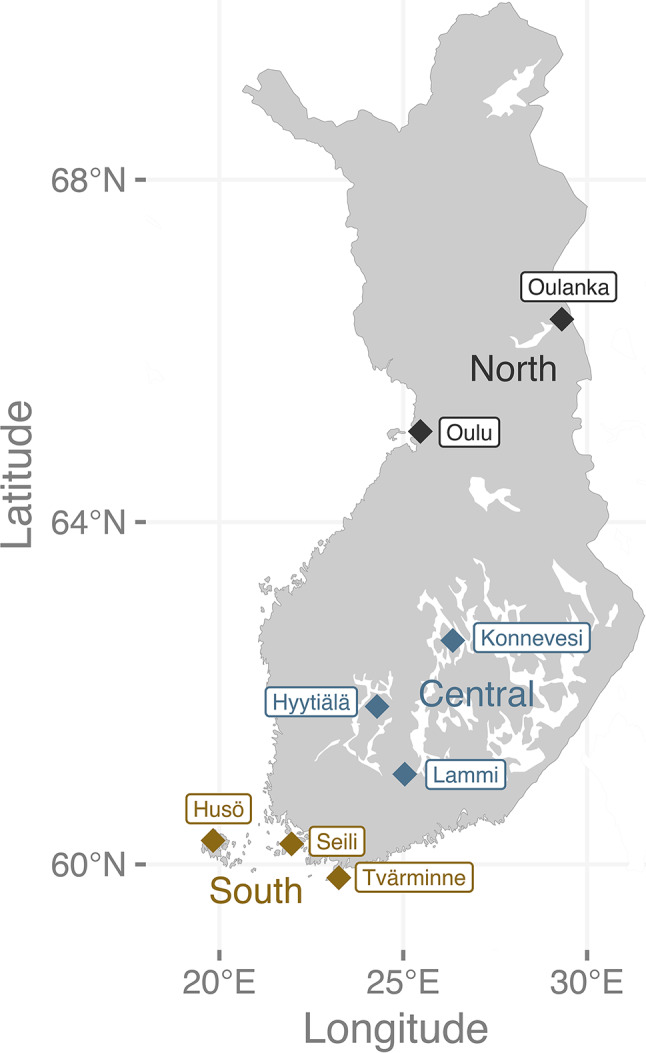
Map of the research stations where data were collected across Finland. We considered the stations to belong to one of three regions: south (yellow), central (blue) or north (black)

A wide-scale passive acoustic monitoring network was initiated in 2015, collecting data every night from May to September at eleven biological research stations across Finland. Data presented here were collected at eight of the stations (in Tvärminne, Seili, Husö, Lammi, Hyytiälä, Konnevesi, Oulu and Oulanka; Fig. 1) yearly until 2021; data from the three northernmost stations (Muddusjärvi, Kilpisjärvi and Kevo) were excluded due to few recordings. All research stations are situated in rural settings, with habitat varying from mixed and broadleaf -forest in Tvärminne to boreal coniferous forest in Oulanka. Two to three SM2BAT + recorders with SMX-U1 ultrasonic microphones (Wildlife Acoustics, Maynard, Massachusetts) were deployed at each station (total number of devices = 23), within 10 m from water bodies and forest edges, with minimal influence of artificial lighting. The units were programmed through Song Meter Configurator (Wildlife Acoustics, Maynard, Massachusetts) to record from 30 min prior to sunset until 30 min after sunrise each night. The sample rate was set at 192 000, with a high pass filter of 16 kHz, a trigger at 18 dB, and a maximum recording length of 10s.

Timing of sunset and sunrise for each night at each location was obtained through the R-package suncalc [41]. We downloaded data of hourly ambient temperature (T_a_), total hourly rainfall (mm), and mean hourly windspeed (m/s) from the closest meteorological stations (from 0.1 km to 34 km away; mean distance: 10.5 km ± 12.4 *SD*) from the Finnish Meteorological Institute (https://en.ilmatieteenlaitos.fi). From these data we calculated nightly (i.e. between sunset to following sunrise) conditions (mean nightly temperature, total nightly rainfall and mean nightly wind-speed). We did not adjust for differences in sampling length for nights with different durations, which could influence the effects of total rainfall.

### Identification of bat calls

We used automated species identification through SonoChiro 4.0 (Biotope, Mèze, France) to detect echolocation calls by our focal species, *E. nilssonii*. We set the classifier to Northern Boreal (NB), call minimum duration to 0.5 s, sensitivity to ten, and specified a minimum number of two calls per recording. Manual checks of a randomly selected sample (*n* = 33) of recordings were used as a quality control of the automated identification, which proved to have a precision of > 95%.

### Statistical analysis

Statistical analyses were performed in the software R 4.4.2 [42]. For all analyses, we used activity minutes (total minutes with recorded activity of *E. nilssonii*) as a quantification of bat activity in this study.

### Proportion of activity across nightly quartiles on ‘active’ nights

To investigate how *E. nilssonii* utilise the available time (time between sunset and sunrise) across varying night lengths and temperature conditions, we divided each night into six periods; one for activity observed before sunset (BSS), four for quartiles between sunset to sunrise (Q1-Q4, each covering 25% of the night), and one for activity observed after sunrise (ASR). For each night we calculated the proportion of activity minutes observed in each period (i.e., the total number of activity minutes recorded within a period divided by the total number of activity minutes recorded for the whole night). Nights with less than 10 activity minutes in total (4 747 nights out of 9 283 nights) were excluded from these analyses, thus making this an investigation of ‘active’ nights. We initially constructed a linear mixed model with proportion of nightly activity as the response variable, explained by three-way interactions between nightly period (BSS, Q1, Q2, Q3, Q4, ASR), night length (h) and, respectively, mean nightly temperature (°C), total nightly rainfall (mm) and mean nightly wind-speed (m/s). Station ID, recorder ID and date of night were included as random effects, with a station: recorder: date nested structure to account for non-independence between observations. However, despite the large number of observations (n_obs_ = 27 216; n_date_ = 964; n_recorder_ = 23; n_station_ = 8), the model failed to converge with any combination of the random effects, indicating too little residual variance left to be explained by these variables in the model. We therefore fitted a simple linear model to the data. We specified Q1 as the reference level for the estimates given our interest in effects relative to the first quartile of the night, as we expected this to generally be the most active nightly period for *E. nilssonii*.

### Total hourly activity across all nights

We further investigated how the total number of activity minutes per hour was related to time since sunset, also including nights with low activity levels (minimum total nightly activity minutes = 1). We specified 13 periods, one for activity observed before sunset (BSS), and 12 for each hour since sunset (period 1 covering activity from sunset until 1 h after sunset, etc.). The data contained observations from nights as long as 15.5 h in duration; however, we excluded periods later than 12 h after sunset from the model given the low number of nights with these periods included and initial investigations indicating very low activity during these hours in general. Time since sunset was divided into hourly groups rather than treated as a continuous variable due to our interest in estimating time-specific effects of environmental conditions throughout the night. We constructed a linear mixed model with log-transformed hourly activity (number of activity minutes per hourly group, adding 1 to each value to avoid 0-observations for the log-transformation) as the response variable, explained by three-way interactions between nightly period (BSS and 1–12 h), night length and, respectively, mean nightly temperature, total nightly rainfall and mean nightly wind-speed. Station ID, recorder ID and date of night were included as random effects with a station: recorder: date nested structure. We specified the first hour after sunset (H1) as the reference level for the estimates.

### Activity across seasons and regions

Because variation in environmental variables like night length and temperature are driven by season and latitude, we were also interested in the overall temporal and spatial variation in nightly activity patterns in our study system. Based on seasonal behavioural patterns in high-latitude *E. nilssonii*, we defined ‘spring’ as the period from winter emergence to mean parturition date in *E. nilssonii* (1. April to 20. June; [43], ‘summer’ as the period from the mean parturition date to the beginning of the pre-hibernation fattening phase (21. June to 28. August; [30, 44], and ‘autumn’ as the period from the pre-hibernation fattening to the beginning of winter hibernation (29. August to 1. November; [30, 44]). We further grouped the eight research stations into three regions; south (Tvärminne, Seili and Husö, from 59.8°N to 60.3°N), central (Lammi, Hyytiälä and Konnevesi, from 61.1°N to 62.6°N), and north (Oulu and Oulanka, from 65.1°N to 66.4°N; Fig. 1). We first constructed a linear mixed model with proportion of nightly activity as the response (only including nights with > 10 activity minutes in total), explained by a three-way interaction between nightly quartiles, season (spring, summer, autumn) and region (south, central, north). However, the model failed to converge with any combination of the random effects (station ID, recorder ID and date). We therefore fitted a linear model to the data. We specified Q1:south: summer as the reference level.

To investigate the total activity across hours, we constructed a linear mixed model with log-transformed hourly activity (number of activity minutes per hourly group, adding 1 to each value to avoid 0-observations for the log-transformation) as the response variable, explained by three-way interactions between nightly period (BSS and 1–12 h), season and region. Station ID, recorder ID and date of night were included as random effects with a station: recorder: date nested structure. We specified H1:south: summer as the reference level for the estimates.

## Results

We recorded *E. nilssonii* activity on a total of 9 283 detector-nights across the seven years, eight research stations and 23 devices in our study.

### Proportion of activity across nightly quartiles on ‘active’ nights

**Fig. 2:**
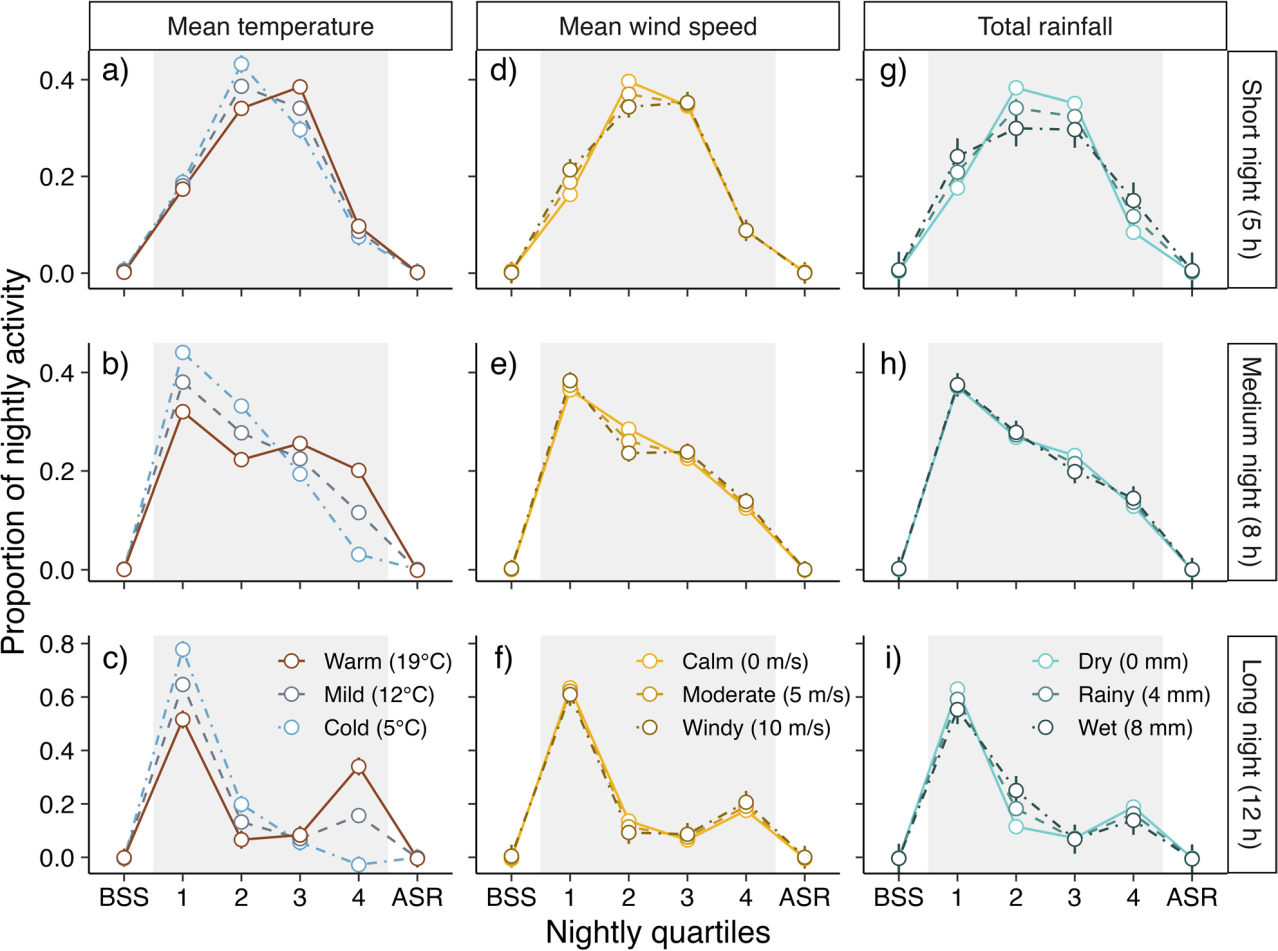
Proportion of activity across nightly quartiles on ‘active’ nights (i.e., nights with 10 or more activity minutes in total). Activity before sunset is shown in the group ‘BSS’, while activity after sunrise is included in the group ‘ASR’. Datapoints and error bars are predictions based on the model output (Table S1), visualising the interaction effect between nightlength (rows) and variation in temperature (**a**-**c**), wind speed (**d**-**f**) and rainfall (**g**-**i**) on nightly activity, with different colours and line-types indicating the effects on nights with varying environmental condition

Activity patterns of *E. nilssonii* across quartiles on ‘active’ nights (detector-nights with more than 10 recorded activity minutes; *n* = 4 536) was strongly influenced by night length, temperature and their interaction, while nightly mean windspeed and total nightly rainfall had a negligible effect on these activity patterns (Fig. 2, Table S1). Figure 2 shows predictions of activity for three different night lengths (5-hour, 8-hour and 12-hour nights) for three nightly temperature scenarios (5 °C, 12 °C and 19 °C; Fig. 2a-c), three windspeed scenarios (0 m/s, 5 m/s and 10 m/s; Fig. 2d-f), and three rainfall scenarios (0 mm, 4 mm and 8 mm; Fig. 2e-i), constructed from the model estimates (Table S1) to illustrate the three-way interaction effects. On short nights, bats were most active during the middle of the night (Q2-Q3) when the sun is at its lowest behind the horizon, with warmer temperatures slightly skewing the activity towards the third quartile (Fig. 2a). With increasing night length, activity generally peaked during the first quartile (Q1), followed by a decline in activity on cold nights (Fig. 2b-c). On warm and intermediate long nights, bats spread out their activity (Fig. 2b), but when nights became very long, the activity decreased during the middle of the night before it increased again in the last part of the night (Q4), resulting in a second nightly activity peak (Fig. 2c). This double peak in activity only became apparent in the long (12-hour) and warm (19 °C) model scenario (Fig. 2c). Windier conditions had negligible effects on activity across nights of different lengths (Fig. 2d-f). Increasing rainfall weakly impacted activity on shorter nights (Fig. 2g), resulting in a slightly more distributed activity-pattern on wetter nights, but had otherwise negligible effect on the proportion of activity across quartiles (Fig. 2h-i).

### Total hourly activity across all nights

**Fig. 3:**
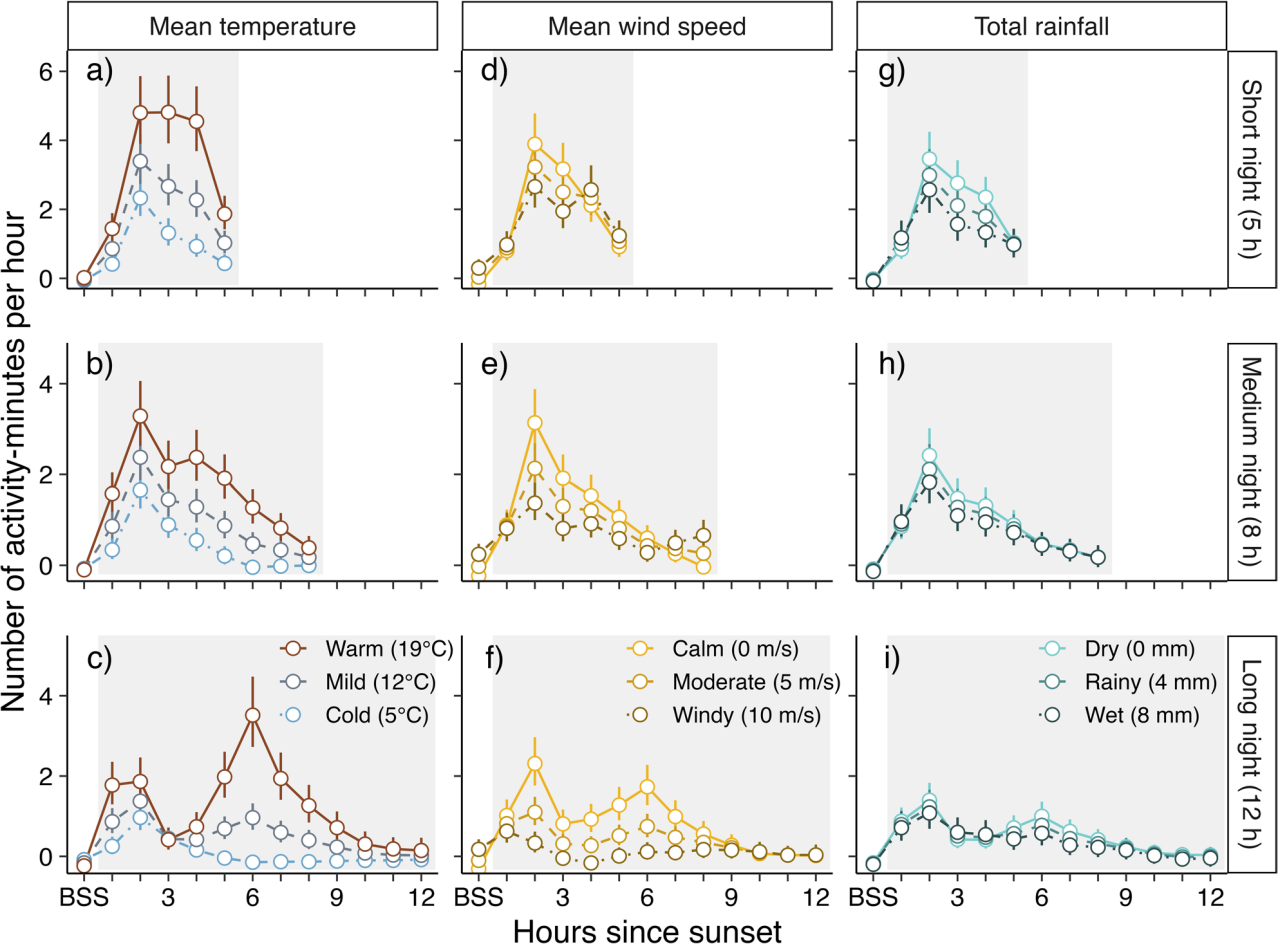
Total hourly activity across all nights. Activity before sunset is shown in the group ‘BSS’. Datapoints and error bars are predictions based on the model output (Table S2), visualising the interaction effect between nightlength (rows) and variation in temperature (**a**-**c**), wind speed (**d**-**f**) and rainfall (**g**-**i**) on nightly activity, with different colours and line-types indicating the effects on nights with varying environmental conditions

Hourly activity of *E. nilssonii* (detector-nights; *n* = 9 283) generally peaked 1–2 h after sunset (H2), but was strongly impacted by night length, temperature and windspeed (Fig. 3, Table S2). Figure 3 shows predictions of hourly activity (back-transformed from log-transformed observations) for three night length scenarios, three temperature scenarios, three windspeed scenarios, and three rainfall scenarios, constructed from the model estimates (Table S2) to illustrate the three-way interaction effects. Overall hourly activity increased with increasing nightly temperature (Fig. 3a-c) and decreasing windspeed (Fig. 3d-f), while increasing rainfall only had a weak negative effect on activity on shorter nights (Fig. 3g). On colder nights, bat activity was always highest 1–2 h after sunset (H2) before quickly tapering off, regardless of night length (Fig. 3a-c). However, with increasing nightly temperature, night length strongly influenced activity patterns; on short, warm nights, activity was spread out across the few available hours of nighttime (Fig. 3a), while increasing night length led to a stronger peak in activity in the early hours of warm nights, before also a second peak appeared 5–6 h after sunset (H6) on the longest nights (Fig. 3c). This dual-peak pattern was also somewhat affected by wind-conditions, with stronger peaks more present on calmer, long nights (Fig. 3f). On shorter nights, increasing windspeed resulted in the hourly activity being more spread out throughout the night, although overall activity decreased (Fig. 3d). Across night lengths, activity was more skewed towards the early hours on calmer nights (Fig. 3d-f). Although rainier nights had somewhat higher overall activity levels on short nights (Fig. 3g), the pattern of activity did not change with rain conditions across night lengths (Fig. 3g-i).

### Activity across seasons and latitudes

**Fig. 4:**
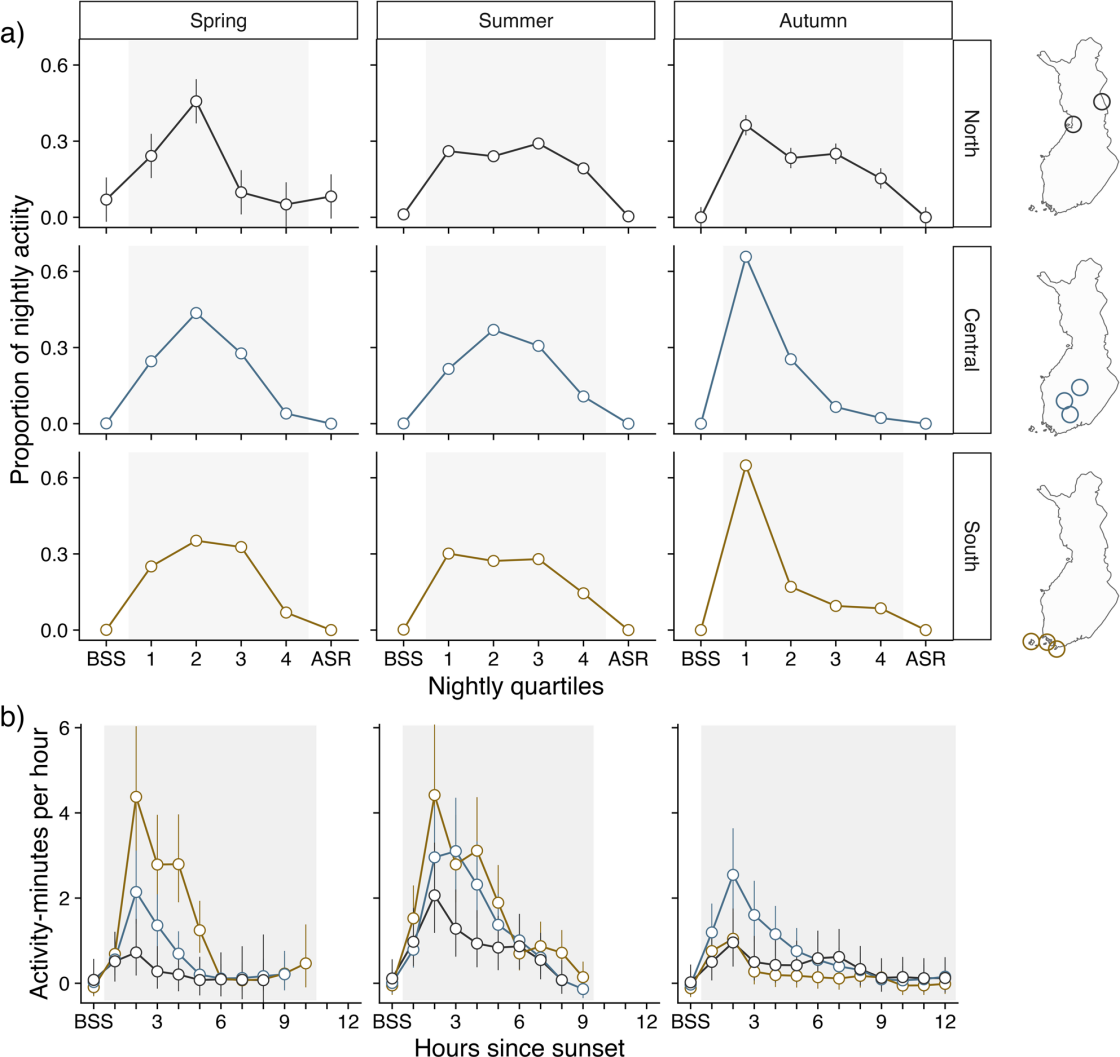
Activity across seasons and regions. Datapoints and error bars are predictions based on the model output (Table S3 and Table S4). Colours indicate different regions (south in yellow, central in blue, and north in black). **(a)** Predicted proportion of activity across nightly quartiles on ‘active’ nights for each season (columns) and regions (rows and colours). **(b)** Predicted hourly activity for each season (columns) and region (colours)

Nightly activity patterns for *E. nilssonii* on ‘active’ nights (detector-nights; *n* = 4 536) across a larger temporal and spatial scale revealed that the use of the available night time generally changed more with season than latitude, although both factors significantly impacted activity across the nightly quartiles (Fig. 4a, Table S3). Predicted activity patterns based on the model estimates (Table S3) are shown in Fig. 4a. In spring, activity peaked in the second quartile of the night (Q2), a pattern that became more evident with increasing latitudes. In summer, the activity across all three regions was more evenly distributed across the night. Activity patterns in autumn, however, were more dependent on latitude, with a strong activity peak in the first quartile of the night (Q1) in the south and central region, with a more evenly distributed activity pattern in the north region.

The hourly activity (detector-nights; *n* = 9 283) showed that the overall activity across all nights decreased with increasing latitude in spring and summer but was lowest in the south region during autumn (Fig. 4b, Table S4). The activity patterns indicated a general peak during H2 (1–2 h after sunset) across all seasons and latitudes.

## Discussion

The results of our study, based on an extensive dataset collected across seven years at several latitudes in the boreal zone, show distinct nightly activity patterns in *E. nilssonii* dependent on night length and nightly weather conditions. During short nights, the highest proportion of activity corresponds to the darkest time of night (Q2-Q3) across all modelled temperature, rainfall and windspeed scenarios, whereas with increasing night length the activity pattern gets strongly influenced by ambient temperature conditions. On longer cold nights, activity is most heavily skewed to the first quartile of the night. With increasing temperature, the activity becomes temporally more evenly distributed throughout the night as night length increases, until a second activity pattern occurs on the longest, warmest nights, with a dual activity-peak in the first and fourth quartile. The model also predicted that overall hourly bat activity increases with ambient temperature and decreasing windspeed across all scenarios of night length, while increasing rainfall only appeared to impact activity on short nights. When the data were modelled across latitudes, the identified patterns reflected the expected prevailing conditions at sampling locations.

In the subarctic climate of the boreal zone, nightly activity patterns of insectivorous bats should be expected to largely track energetically profitable foraging conditions while also reducing individual predation risk [1]. We predicted that *E. nilssonii* activity on cold nights would be low and limited to the time immediately following sunset, which would reflect the activity patterns of crepuscular insects [12]. Although these predictions held true across all night lengths modelled, a peak in activity during the second hour after sunset was observed in all modelled scenarios. The fact that this peak is evident even on nights that would be considered warm and mild in temperature, where the thermoregulatory costs of remaining active to forage would be low, suggests that insect availability is likely driving this temporal pattern of foraging activity.

On short nights, the peak in activity after sunset is not as prominent, particularly if examining activity patterns as nightly quartiles. This would lead to the assumption that due to the short duration of the night, the entire night can be considered to fall within the extent of the activity peak. However, inspecting hourly total activity reveals that instead of the activity peaking 1–2 h after sunset (H2 in Fig. 3) for a single hour only as in the medium- and long night scenarios, the activity during short nights is almost uniform for three hours on warm nights in particular. This suggests bats effectively forage throughout the night when the nights are at their shortest. This may well reflect the life history of *E. nilssonii*, in which parturition, and ensuing lactation, take place at around summer solstice [39], placing female bats under higher energetic needs in which foraging time is maximised at the cost of decreased night-time roosting [12].

In our dataset, a specific model scenario of long, warm nights resulted in a predicted second activity peak in the fourth nightly quartile. The shift in activity patterns from the unimodal (activity peak at dusk) to bimodal (activity peaks at both dusk and dawn) has previously been reported for some aerial-hawking bat species in Europe [2, 4]. Modality (unimodal versus bimodal) in nightly activity patterns in bats can depend on species [3–5] but can also vary within species depending on reproductive period [2, 6–9], landscape characteristics [5, 10] and sex or age [6, 45]. However, in order for a second activity peak to occur, the expected foraging profit needs to outweigh the energetic expenditures of foraging. Hawking bats that express bimodal activity patterns have been shown to exploit dual activity peaks of aerial insects after dusk and before dawn [2, 46]. Here, we show that a specific combination of long and warm nights is required for a second activity peak to be observed in our dataset. If nights are warm, but short, the available time to forage is limited while aerial insect activity is likely high throughout the night, resulting in a more spread-out activity pattern for *E. nilssonii*. On longer nights, dawn-activity appears to be profitable only during warmer conditions.

The effects of temperature in shaping the distribution of insect abundance between nights rely on the notion that activity in these ectothermic organisms are broadly thermally constrained [47]. The physiological performance of ectotherms increases with temperature until reaching a peak after which physiological condition rapidly declines as suggested by the thermal performance theory [48]. With environmental temperatures peaking during the day, higher maximum environmental temperatures may select for increased nocturnality in insect communities as more individuals avoid heat stress from daytime temperatures that approximate their upper thermal limits [31, 49]. This most likely does not have a significant influence on insect activity at northern latitudes, but nocturnal activity still allows insects to avoid diurnal, visual-hunting predators [49]. However, with increasing latitude, lower temperatures during the night in the boreal zone may reach the lower thermal threshold of insects thereby limiting activity after the crepuscular period [31, 49]. Indeed, a large proportion of the diet of boreal bats, and even *E. nilssonii*, in particular, contain nocturnal taxa that are active already during the crepuscular period, such as non-biting midges and geometrid moths (Chironomidae and Geometridae [50–52]). Further studies are needed to better understand the diel patterns and thermal constraints of aerial insect taxa at northerly latitudes to allow a better understanding of nightly- and seasonal bat activity patterns. This need is particularly pressing with evidence of anthropogenic environmental change causing not only general insect decline [53], but also a shift in insect community structure, which may greatly their influence activity patterns with carry-on effects on insectivore communities [54].

As predicted, and in line with existing research [8, 27, 55], overall bat activity increases with improving nightly weather conditions (warmer, calmer or drier nights), although the effect of precipitation is mainly evident on short nights. With increasing windspeed, the reduced activity is also more spread out across the night-time hours. This pattern could indicate that bats foraging during sub-optimal weather conditions (i.e., higher wind speed) are more prone to take advantage of rapid shifts in environmental conditions during the night. Previous studies at high latitudes show bats supressing activity during rain showers, but resuming foraging whenever precipitation tapered off, with similar observations existing in the tropics [56, 57]. However, to predict hourly responses to temporal fluctuations in wind- or rain conditions during the night, environmental data would need to be collected on a finer timescale than overall nightly conditions.

Finally, we used our empirical dataset to translate our model to potential scenarios over the active season of bats at varying latitudes across Finland. The observed patterns reflect the varying climatic conditions at each latitude, with the largest contrasts occurring between the southernmost (60°) and northernmost (65°) latitudes. The most frequent nightly pattern is the “short night” pattern in which no clear peak in activity is seen during the second hour after sunset, but rather a more uniform or bell-shaped activity curve across the night. Signs of the bimodal pattern are observed only at the most southerly location of our study as nights grow longer but still remain relatively warm in the autumn. Further studies are needed to better understand how species-specific life history traits may influence these observed patterns along photoperiod and climatic conditions in the boreal and sub-arctic bat fauna.

Our work builds on previous studies linking abiotic conditions to animal activity but advances the field by integrating our unique, long-term, large-scale monitoring with fine-scale behavioral analysis. It contributes to a broader understanding of how general ecological principles—such as optimal foraging, temporal niche partitioning, and behavioral plasticity—play out in extreme seasonal environments. Being able to identify these spatiotemporal patterns emphasizes the importance of large-scale and long-term datasets, such as utilised in the current study, with similar results achievable using citizen science approaches [58, 59]. These allow the effective monitoring of the impacts of climate change on not only the activity patterns and abundance of bats across latitudes, but also identifying changes in species composition and relative abundance [60, 61]. However, considering the rate of current global change, future studies need to better understand drivers of both daily and seasonal insect patterns [62], which directly contribute to patterns observed in bat fauna.

## Supplementary Information

Below is the link to the electronic supplementary material.

Supplementary Material 1

## Data Availability

The dataset has been uploaded to the online data repository Zenodo. DOI: 10.5281/zenodo.18298435.
